# Pharmacokinetic Evaluation of Metabolic Drug Interactions between Repaglinide and Celecoxib by a Bioanalytical HPLC Method for Their Simultaneous Determination with Fluorescence Detection

**DOI:** 10.3390/pharmaceutics11080382

**Published:** 2019-08-02

**Authors:** Dong-Gyun Han, Jinsook Kwak, Seong-Wook Seo, Ji-Min Kim, Jin-Wook Yoo, Yunjin Jung, Yun-Hee Lee, Min-Soo Kim, Young-Suk Jung, Hwayoung Yun, In-Soo Yoon

**Affiliations:** 1 Department of Manufacturing Pharmacy, College of Pharmacy, Pusan National University, Busan 46241, Korea; 2 Department of Pharmacy, College of Pharmacy, Seoul National University, Seoul 08826, Korea

**Keywords:** celecoxib, drug–drug interaction, fluorescence, HPLC, metabolism, repaglinide

## Abstract

Since diabetes mellitus and osteoarthritis are highly prevalent diseases, combinations of antidiabetic agents like repaglinide (REP) and non-steroidal anti-inflammatory drugs (NSAID) like celecoxib (CEL) could be commonly used in clinical practice. In this study, a simple and sensitive bioanalytical HPLC method combined with fluorescence detector (HPLC-FL) was developed and fully validated for simultaneous quantification of REP and CEL. A simple protein precipitation procedure and reversed C18 column with an isocratic mobile phase (mixture of ACN and pH 6.0 phosphate buffer) were employed for sample preparation and chromatographic separation. The fluorescence detector was set at a single excitation/emission wavelength pair of 240 nm/380 nm. The linearity (10–2000 ng/mL), accuracy, precision, extraction recovery, matrix effect, and stability for this method were validated as per the current FDA guidance. The bioanalytical method was applied to study pharmacokinetic interactions between REP and CEL in vivo, successfully showing that concurrent administration with oral REP significantly altered the pharmacokinetics of oral CEL. Furthermore, an in vitro metabolism and protein binding study using human materials highlighted the possibility of metabolism-based interactions between CEL and REP in clinical settings.

## 1. Introduction

Arthritis and diabetes mellitus are highly prevalent diseases with a total of over 350 million patients worldwide [1,2]. The most common types of arthritis and diabetes mellitus are osteoarthritis (OA) and type 2 diabetes mellitus (T2DM), respectively [3]. OA affects 14% of adults aged ≥25 years, and 34% of these patients are aged >65 years [4]; similarly, T2DM affects 12% of adults aged ≥20 years, and 26% of these are aged >65 years [5]. A recent survey estimated that the prevalence of OA was higher in individuals with T2DM than in those without T2DM [6]. Thus, T2DM is generally recognized as a comorbidity of arthritis [7], while some previous studies have focused on diabetes as a risk factor of arthritis [8,9]. Anyway, it is evident that T2DM is closely associated with an increased incidence and prevalence of OA, though the reasons remain unclear.

**Figure 1:**
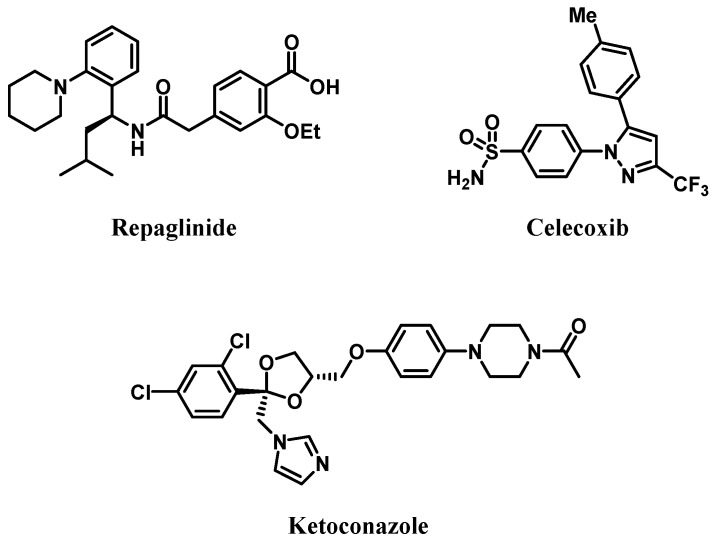
Chemical structures of repaglinide, celecoxib, and ketoconazole (internal standard, IS).

Repaglinide (REP), as shown in Figure 1, is a short-acting oral antidiabetic drug belonging to the class of meglitinides and is used to lower postprandial blood glucose levels in T2DM patients [10]. It stimulates insulin release from the pancreas, depending on the residual function of β-cells in the pancreatic islets [11]. REP is eliminated primarily by CYP2C8- and CYP3A4-mediated oxidative metabolism [12]. Systemic exposure to REP has been reported to be altered by co-administration of trimethoprim (inhibitor of CYP2C8) [13], itraconazole (inhibitor of CYP3A4) [14], or rifampicin (inducer of CYP3A4) [11]. Celecoxib (CEL), as shown in Figure 1, is a cyclooxygenase-2 (COX-2) selective non-steroidal anti-inflammatory drug (NSAID) and is used to treat pain and inflammation associated with OA and rheumatoid arthritis (RA) [15]. Because gastrointestinal mucosal integrity is compromised by COX-1 inhibition, traditional nonselective NSAIDs such as aspirin, ibuprofen, and indomethacin that inhibit both COX-1 and COX-2 may cause serious side effects in the gastrointestinal tract [16]. Thus, CEL exhibits distinctly reduced gastrointestinal toxicity as compared to conventional NSAIDs, thereby becoming a blockbuster drug for treating OA and RA [17]. CEL is eliminated primarily by extensive metabolism through methyl hydroxylation to form hydroxycelecoxib, which is catalyzed by CYP2C9 and CYP3A4 [18].

Because T2DM frequently co-exists with OA, there is a possibility of concurrent administration of REP and CEL. Hence, a bioanalytical method of simultaneous determination of REP and CEL could be useful and efficient for further pharmaceutical development and therapeutic optimization. To date, several bioanalytical methods have been developed and validated for quantitative determination of REP or CEL individually using HPLC with UV/Vis detection [19,20,21,22,23] or using liquid chromatography with tandem mass spectrometry (LC-MS/MS) systems [24,25,26]. However, these methods are associated with a few limitations, such as an insufficient sensitivity, relatively large sample volume, and/or time-consuming liquid–liquid extraction procedures with volatile solvents that are potentially hazardous to health. Moreover, LC-MS/MS methods require relatively complex and/or expensive instrumentation, which may not be affordable for small-sized laboratories and companies in resource-limited settings. To the best of our knowledge, there have been no reported methods of simultaneous quantification of REP and CEL in biological samples using HPLC coupled with a fluorescence detector (HPLC-FL). Furthermore, a previous in vitro study reported that CEL inhibited REP metabolism in pooled human liver microsomes (HLM) with a K_i_ of 3.1 μM [27]. This suggests the possibility of pharmacokinetic drug interaction between REP and CEL, but no information is currently available regarding this issue. Therefore, further investigation of the pharmacokinetic drug interaction between REP and CEL is necessary to prevent adverse effects in the use of these drugs.

In the current study, a sensitive and simple HPLC-FL method was developed and fully validated for simultaneous quantification of REP and CEL in rat plasma. The linearity, sensitivity, precision, accuracy, recovery, matrix effect, and stability of this HPLC-FL method were determined. Next, the potential for the pharmacokinetic drug interactions between REP and CEL was investigated in vivo using Sprague-Dawley rats and in vitro using rat liver microsomes (RLM) and HLM.

## 2. Materials and Methods

### 2.1. Materials

CEL (purity ≥98%), ketoconazole (as internal standard [IS]; purity ≥98%), as shown in Figure 1, and REP (purity >98%) were purchased from Tokyo Chemical Industry Co. (Tokyo, Japan). DMSO, ethanol, potassium phosphate monobasic/dibasic, and polyethylene glycol 400 were purchased from Sigma-Aldrich Co. (St. Louis, MO, USA). β-nicotinamide adenine dinucleotide phosphate (NADPH), HLM, and RLM were purchased from BD-Genetech (Woburn, MA, USA). ACN and methanol of HPLC grade were purchased from Thermo Fisher Scientific, Inc. (Waltham, MA, USA).

### 2.2. Animals

Male Sprague-Dawley rats (nine-week-old; approximately 300 g) were purchased from Samtako Bio Korea Co. (Gyeonggi-do, Korea). They were kept in a clean room of the Laboratory Animal Center of Pusan National University (Busan, Korea) at a relative humidity of 50 ± 5% and temperature of 20–23 °C with 12 h dark (19:00–07:00) and light (07:00–19:00) cycles. They were housed in metabolic cages (Tecniplast USA Inc., West Chester, PA, USA) with tap water and standard chow diet (Agribrands Purina Canada Inc., Levis, QC, Canada) provided ad libitum. The present animal study protocols were approved by the Pusan National University-Institutional Animal Care and Use Committee (PNU-IACUC, Busan, South Korea) for ethical procedures and scientific care (approval number: PNU-2018-1848; approval date: 01/05/2018).

### 2.3. Calibration Standards and Quality Control Samples

Stock solutions of REP, CEL, and IS (1000 μg/mL in DMSO) were prepared. The stock solutions of the mixture of REP and CEL were diluted with mobile phase for the preparation of working standard solutions with concentrations ranging from 1 to 200 μg/mL. The working solution of IS (final concentration of 5 μg/mL in ACN) was prepared by diluting the stock solution of IS with ACN. Calibration standard samples were prepared by spiking blank rat plasma with each working solution, yielding final plasma concentrations of 10, 20, 50, 100, 200, 500, 1000, and 2000 ng/mL. Quality control (QC) samples were prepared from separate stocks of REP and CEL in an identical manner to the preparation of calibration standards. The concentration levels of QC samples were 10 (lower limit of quantification; LLOQ), 30 (low; LQC), 120 (middle; MQC), and 1200 ng/mL (high; HQC).

### 2.4. Sample Preparation

For deproteinization, 400 μL ice-cold ACN containing IS (50 ng/mL) was added to 120 μL plasma samples. The resultant mixture was vortex-mixed for 5 min, followed by centrifugation at 15,000× *g* for 5 min. Next, 400 μL supernatant was transferred to another microtube and dried by N_2_ gas stream. For reconstitution, 60 μL mobile phase was added to the resultant residue, and after sufficient vortex-mixing, 20 μL finally prepared sample solution was injected to the HPLC system.

### 2.5. Chromatographic Conditions

In this study, we used a Shimadzu HPLC system (Shimadzu Co., Kyoto, Japan) equipped with a fluorescence detector (RF-20A), column oven (CTO-20A), autosampler (SIL-20AC), and pump (LC-20AT). A Kinetex C18 column (250 × 4.6 mm, 5 μm, 100 Å; Phenomenex, Torrance, CA, USA) protected by a C18 guard column (SecurityGuard HPLC Cartridge System; Phenomenex) at 40 °C was used for chromatographic separation. Isocratic elution of mobile phase consisting of 10 mM phosphate buffer (pH 6.0) and ACN (53.6:46.4, *v*/*v*) was performed at a flow rate of 1 mL/min. The injection volume and total run time were 20 μL and 23 min, respectively. The fluorescence excitation and emission wavelengths for REP, CEL, and IS were 240 and 380 nm, respectively.

### 2.6. Method Validation

This new bioanalytical method for simultaneous determination of REP and CEL was validated based on the US-FDA guidelines [28]. The selectivity was assessed based on the comparison among chromatograms of REP, CEL, and IS in blank rat plasma; blank rat plasma spiked with REP, CEL, and IS; and rat plasma sample obtained from a pharmacokinetic study in rats. The presence of endogenous interferences at the acquisition windows of the analytes was examined.

The linearity was determined by the addition of increasing amounts of REP and CEL to a blank biological matrix. Calibration curves (*n* = 5) were constructed by plotting the peak area ratios of analytes to IS (*y*-axis) versus the concentration ratios of REP and CEL (10–2000 ng/mL) to IS (50 ng/mL) in plasma (*x*-axis), and linear regression analysis was conducted using the least squares method with a weighting factor of 1/*x* (*x* = concentration). The sensitivity was assessed based on LLOQ, defined as the lowest quantifiable concentration levels of REP and CEL in calibration curves (signal-to-noise [S/N] ratio of more than 5). REP and CEL peaks at the LLOQ level should be identifiable, reproducible, and discrete with acceptable accuracy (within 80–120%) and precision (<20%).

The precision and accuracy were estimated by comparison between the measured concentrations and their respective nominal concentrations in the QC samples, which were prepared as five separate sets on one day (intra-day) and five different days (inter-day). Precision was expressed as a coefficient of variation (CV) of the mean values of the measured concentration. Accuracy was expressed as a relative error between the measured and nominal concentrations. They were determined with plasma samples spiked with REP and CEL at the four different QC levels in five replicates.

The extraction recovery and matrix effect were determined by comparison among the analytical signals (peak area) obtained from (A) the extracted sample, (B) the post-extracted spiked sample (extracts of blanks spiked with the analyte post extraction), and (C) non-extracted neat sample (diluted stock solution). The recovery was calculated as ‘A/B × 100’, and the matrix effect was calculated as ‘B/C × 100’. Five replicates were assessed at the four different QC levels.

The stability was assessed by comparison of the analytical signals (peak area) obtained from plasma samples exposed to various handling and storage conditions with those obtained from plasma samples. Bench-top stability was determined by exposing spiked plasma samples to room temperature for 180 min. Freeze–thaw stability was determined by exposing spiked plasma samples to freeze–thaw cycles (from −20 °C to room temperature) three times on consecutive days. Long-term stability was determined by storing spiked plasma samples at −20 °C for 30 days. Autosampler stability (post-preparative stability) was determined by exposing extracted plasma samples to 25 °C for 1 day in an autosampler. The stability was determined at the four different QC levels.

### 2.7. In Vivo Pharmacokinetic Study in Rats

Rats were fasted for 12 h prior to the pharmacokinetic experiment and then anesthetized with zoletil (intramuscular, 20 mg/kg). The femoral artery and vein of the rats were cannulated with a polyethylene tube (BD Medical; Franklin Lakes, NJ, USA) at 240 min prior to drug dosing. A single oral dose of REP alone (0.4 mg/kg), CEL alone (2 mg/kg), or REP and CEL at the same doses was administered to the rats (*n* = 5 per group). Drugs were dissolved in a vehicle that is a clear mixture of DMSO, ethanol, polyethylene glycol 400, and saline at a ratio of 1:5:30:64 (*v*/*v*/*v*/*v*). Approximately 300 μL aliquots of blood were collected in heparin pre-treated microcentrifuge tubes via the femoral artery at 0, 10, 20, 30, 45, 60, 90, 120, 180, 240, 360, and 480 min after the oral dosing. Following centrifugation of blood samples at 2000× *g* at 4 °C for 10 min, 120 μL aliquots of plasma were stored at −80 °C until HPLC analysis.

### 2.8. In Vitro Metabolism and Protein Binding Study

An in vitro microsomal metabolism study was conducted using Corning^®^ Gentest^TM^ pooled male RLM (from Sprague-Dawley rats) and HLM (from more than 5 male donors) as previously described [29,30], with slight modifications and in accordance with the manufacturer’s protocol. To assess the possibility of metabolic interaction between REP and CEL, a microsomal reaction mixture was prepared as follows (total volume: 0.2 mL): RLM or HLM (0.5 mg/mL), 50 mM phosphate buffer, 1 mM NADPH, 10 mM MgCl_2_, 1 μM substrate, and various concentrations of inhibitor (1–100 μM). The disappearance rates of REP (as a substrate) in the absence or presence of CEL (as an inhibitor), and vice versa, were determined. At 0 and 15 min (REP) or 0 and 45 min (CEL) after starting the metabolic reaction, a 50 μL aliquot of microsomal incubation mixture was sampled and transferred into a clean 1.5 mL microcentrifuge tube containing 100 μL cold ACN containing IS (50 ng/mL) to stop the metabolic reaction. After vortex mixing and centrifugation at 15,000× *g* for 10 min, a 100 μL aliquot of the supernatant was stored at −80 °C until HPLC analysis.

The fractions of unbound REP and CEL (f_u_) in rat and human plasma were measured using the rapid equilibrium dialysis (RED) device (Thermo Fisher Scientific, Inc.) as previously described [31]. The plasma was spiked with REP alone, CEL alone, and both drugs, yielding final concentration of 10 μM. A 0.2-mL spiked plasma was placed into the ‘sample’ chamber, and a 0.35 mL isotonic phosphate buffered saline was placed into the adjacent ‘buffer’ chamber. The fraction unbound was calculated as the ratio of the drug concentrations in the ‘buffer’ compartment to those in the ‘sample’ compartment.

### 2.9. Data Analysis

The IC_50_ of CEL for the inhibition of the metabolism of REP was determined by GraphPad Prism 5.01 (GraphPad Software, San Diego, CA, USA) according to the following Hill equation:

$$y=\mathrm{Min}+\frac{\mathrm{Max}-\mathrm{Min}}{1+{\left(\frac{x}{{\mathrm{IC}}_{50}}\right)}^{-P}}.$$

Analytical data were acquired and processed using the LC Solution Software (Version 1.25; Shimadzu Co.). Non-compartmental analysis was conducted to estimate pharmacokinetic parameters such as total area under plasma concentration versus time curve from time zero to infinity (AUC_inf_), total area under plasma concentration versus time curve from time zero to time of last sampling (AUC_last_), and terminal half-life (*t*_1/2_) using the NCA200 and 201 models of WinNonlin software (Version 3.1; Certara USA Inc., Princeton, NJ, USA) [32]. Peak plasma concentration (*C*_max_) and time to reach *C*_max_ (*T*_max_) were directly read from the observed data.

### 2.10. Statistical Analysis

A *p*-value below 0.05 was considered statistically significant by using *t*-test for comparison between two unpaired means or by using analysis of variance (ANOVA) with post-hoc Tukey’s honestly significant difference test for comparison among three unpaired means. Unless indicated otherwise, all data except *T*_max_ were expressed as mean ± standard deviation (median (ranges) for *T*_max_). All data numbers were rounded to three significant figures.

## 3. Results and Discussion

### 3.1. Method Development

In this study, various chromatographic conditions were evaluated for sufficient sensitivity and good separation of analytes from endogenous substances of biological matrix within an appropriate run time. Several experiments were performed to choose suitable stationary phase, mobile phase, sample preparation procedure, and IS.

To choose a stationary phase, several types of HPLC columns including Accucore^TM^ HILIC LC column (150 × 4.6 mm, 2.6 μm; Thermo Fisher Scientific, Waltham, MA, USA), XTerra Shield RP18 column (150 × 3.9 mm, 5 μm; Waters Co., Milford, MA, USA), and Kinetex^®^ C8 and C18 columns (250 × 4.6 mm, 5 μm; Phenomenex) were tested. Our analysis found that Kinetex^®^ C18 column showed higher peak resolution and intensity than other columns (data not shown). Therefore, Kinetex^®^ C18 column was chosen as a stationary phase for the analytes.

The composition of mobile phase was optimized with different buffer types, such as citrate buffer (pH 3–5) and phosphate buffer (pH 6–7), and various ACN contents. Changes in the pH of mobile phase considerably influenced the peak retention times of REP (acidic compound) and endogenous interferences; however, it exerted little influence on those of CEL and ketoconazole that are neutral compounds. As a result, the mobile phase of pH 6.0 containing 46.4% ACN achieved good separation from endogenous interference in plasma with acceptable peak resolution. Thus, we settled for this mobile phase in developing the present HPLC-FL method.

Sample preparation was performed using a solvent precipitation-reconstitution method which is an efficient and economical sample pretreatment procedure compared with a solid phase or liquid–liquid extraction method. For optimization, several organic solvents, such as acetone, methanol, trichloroacetic acid, ACN, and their mixtures, were evaluated. Among them, ACN yielded the lowest matrix effect and highest recovery for analytes following centrifugation at 15,000× *g* for a relatively short precipitation time of 5 min.

Several fluorescent compounds, such as diclofenac, diflunisal, doxorubicin, metoprolol, naproxen, propranolol, and quinidine, were tested as a potential IS. However, these were unsuitable as IS, due to poor separation from analytes and endogenous substances in biological matrix. As a result, ketoconazole was finally chosen, because it exhibited good separation with acceptable retention time, peak resolution, and fluorescence intensity at the same wavelength as REP and CEL.

### 3.2. Method Validation: Selectivity, Linearity, Sensitivity, Precision, and Accuracy

**Figure 2:**
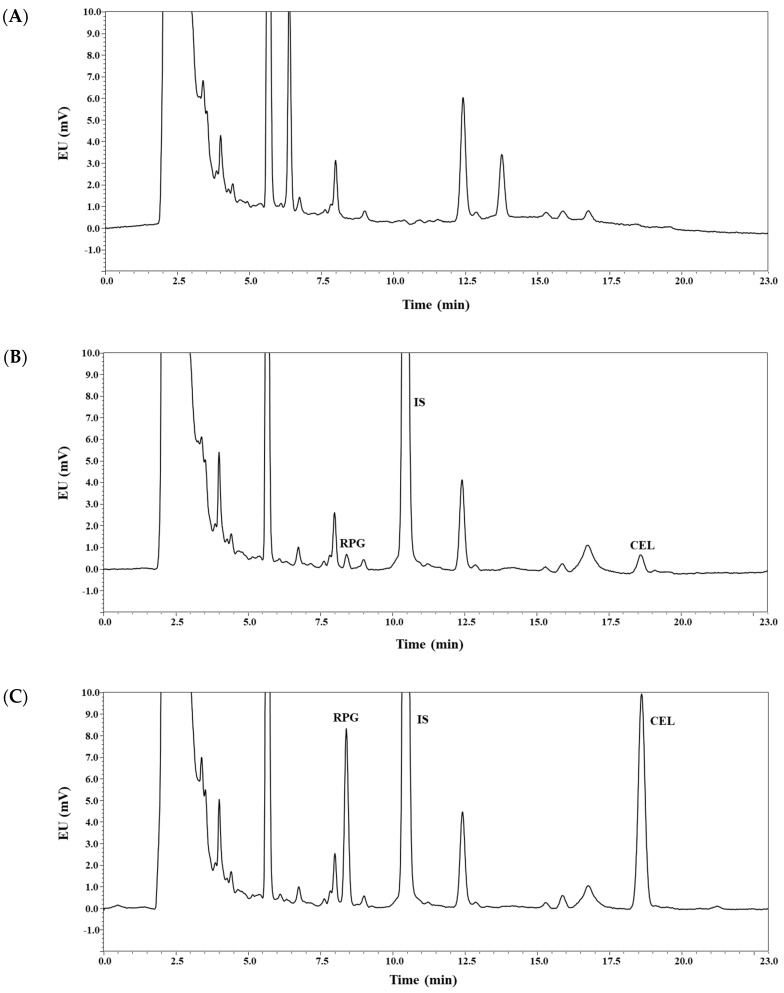

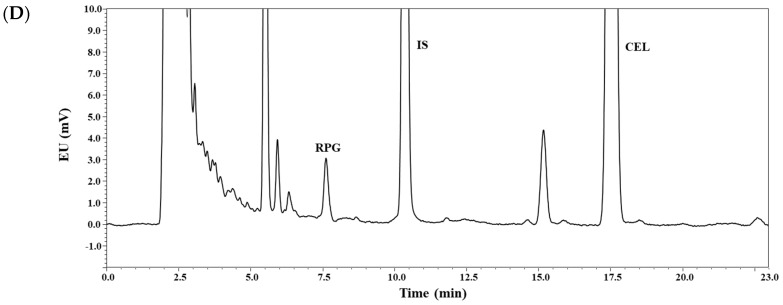
Representative chromatograms of repaglinide (REP), celecoxib (CEL), and ketoconazole (IS) in rat plasma: blank rat plasma (**A**); blank rat plasma spiked with REP, CEL (10 ng/mL, lower limit of quantification (LLOQ)), and IS (**B**); blank rat plasma spiked with REP, CEL (120 ng/mL, middle quality control (MQC)), and IS (**C**); plasma sample collected 120 min after concurrent oral administration of REP and CEL solution in rats, where calculated concentrations of REP and CEL were 53 and 968 ng/mL, respectively (**D**). EU: emission unit.

As shown in Figure 2, the analyte peaks were well separated from each other and from endogenous matrix peaks in the blank plasma. Thus, it appears that the present bioanalytical method could offer acceptable selectivity without endogenous interferences occurring at the retention times of the analytes. The calibration curves (REP-to-IS or CEL-to-IS peak area ratio versus REP or CEL concentration, respectively) for REP and CEL were observed to be linear from 10 to 2000 ng/mL in rat plasma samples. A representative equation for the calibration curves was constructed, as follows: *y* = 1.018*x* − 3.029 for REP and *y* = 2.093*x* − 0.723 for CEL, where *y* represents the ratio of the peak area of REP or CEL to that of IS, and *x* represents the ratio of nominal concentration of REP or CEL. The correlation coefficients (*r*^2^) were over 0.999, showing good linearity of this method. Generally, the sensitivity of a bioanalytical method is represented by the LLOQ value, which, in the present study, was determined to be 10 ng/mL for both REP and CEL. Moreover, the present method offered good sensitivity for CEL, with LLOQ comparable to those reported by previous LC-MS/MS methods in human plasma (LLOQ: 5–10 ng/mL; plasma volume: 100–200 μL) [25,26,33]. The intra- and inter-day precision and accuracy of this method were determined for REP and CEL at the four different QC levels, as shown in Table 1. The precision was estimated to be 8.30% or less, and the accuracy ranged from 98.6% to 112%. These values are within a generally acceptable range, showing that the present method was precise, accurate, and reproducible.

### 3.3. Method Validation: Recovery, Matrix Effect, and Stability

As shown in Table 2, we assessed the recovery and matrix effect of the method for REP and CEL at the four different QC levels and for IS at 50 ng/mL. The mean recovery of REP and CEL was observed to be 98.5–104% with CV values of ≤2.57%. There were no significant differences in recovery values among the four different QC levels (*p* = 0.066 for REP and 0.502 for CEL), indicating concentration-independent recovery for both drugs. The mean matrix effect for REP and CEL was observed to be 92.1–102% with CV values of ≤5.25%. The stability was assessed under various handling and storage conditions relevant to this HPLC-FL method. Bench-top stability, autosampler stability, freeze–thaw stability, and long-term stability were determined for REP and CEL at the four different QC levels. The extent of bias in the concentration was within ±15% of the corresponding nominal value, while the remaining fraction of REP and CEL was observed to be 89.2–106% with CV values of ≤6.04%, as shown in Table 3. These data clearly indicate that the sample preparation procedure employed in the bioanalytical method proposed herein offered sufficient extraction recovery with minimal matrix effect, and that REP and CEL remained stable under several conditions related to the present bioanalytical procedures.

### 3.4. Pharmacokinetic Drug Interaction Studies

**Figure 3:**
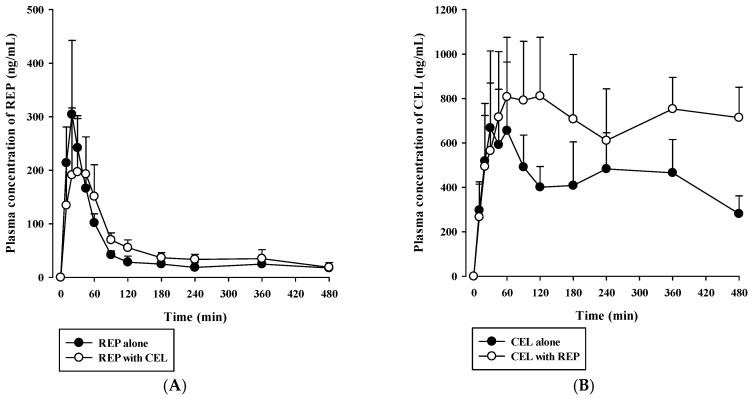
Plasma concentration versus time profiles of REP (**A**) and CEL (**B**) after oral administration of 0.4 mg/kg REP and 2 mg/kg CEL either alone (closed circle) or in combination (open circle) to rats. The circles and vertical bars represent means and standard deviations, respectively (*n* = 5).

Rats received oral REP (0.4 mg/kg) and CEL (2 mg/kg) either alone or in combination. Then, plasma concentration versus time profiles of REP and CEL were evaluated as shown in Figure 3. The relevant pharmacokinetic parameters are listed in Table 4. The oral doses used were selected based on previous rat pharmacokinetic studies on REP or CEL [34,35,36]. After oral dosing of REP, plasma REP levels increased for 20 to 45 min and then declined in a multi-exponential fashion. The AUC_last_, AUC_inf_, and *t*_1/2_ of REP were not significantly changed by concurrent administration with oral CEL, as shown in Table 4. After oral administration of CEL, its plasma concentration profiles markedly fluctuated during the whole period of blood collection (480 min). Thus, the AUC_inf_ and *t*_1/2_ of CEL could not be determined in this study because there was no discernible linear terminal phase observed in the plasma concentration versus time curves of CEL. The multiple peaks in the plasma concentration profiles of CEL may be caused by slow and variable gastrointestinal absorption, which warrants further investigation. Notably, the AUC_last_ of CEL was significantly higher (by 76.2%) after co-administration of CEL and REP than after administration of CEL alone (*p* = 0.0213). Because CEL is eliminated primarily by extensive metabolism [15], the increased systemic exposure of oral CEL could be attributable to a reduction of hepatic first-pass and/or systemic metabolism of CEL caused by concurrent administration of REP.

**Figure 4:**
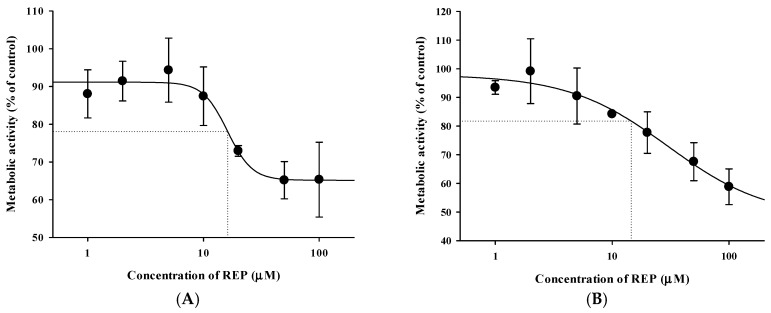
Dose-response curves for the inhibitory effect of REP on metabolic reactions of CEL in RLM (**A**) and HLM (**B**). The circles and vertical bars represent the means and standard deviations, respectively (*n* = 4).

**Figure 5:**
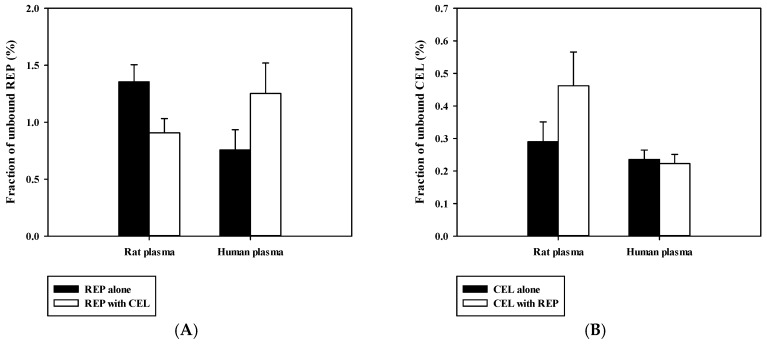
Fraction of unbound REP (**A**) and CEL (**B**) either alone or in combination in rat and human plasma (*n* = 3).

Previously reported pharmacokinetic parameters of intravenous REP and CEL in rats are listed in Table S1. Because the blood-to-plasma concentration ratio (R_B_) was 0.61 for REP and 2.66 for CEL in rat blood (our in-house data), the blood CL (CL_B_) was determined to be 8.52 mL/min/kg for REP and 2.92 mL/min/kg for CEL (calculated as plasma CL/R_B_). Because the urinary excretion of the unchanged drug was reported to be negligible for both drugs, as shown in Table S1, it is plausible to assume that the CL_B_ of REP and CEL could represent their hepatic clearance, which is far below the reported hepatic blood flow rate in rats (Q_H_; ranging from 50 to 80 mL/min/kg) [37]. This indicates that REP and CEL are drugs with low hepatic extraction ratios of 0.037 to 0.170 (calculated as CL_H_/Q_H_). Based on the well-stirred model, the CL_H_ of a drug with a low hepatic extraction ratio primarily depends on its intrinsic metabolic clearance (CL_int_) and fraction unbound in blood (f_B_) [30]. Thus, the hepatic metabolism and plasma protein binding of the drugs were further investigated using an in vitro rat and human liver microsomes and plasma. As shown in Figure 4, dose-response curves for the inhibitory effect of REP on the metabolism of CEL were constructed in RLM and HLM. REP significantly inhibited the metabolic reaction of CEL with IC_50_ values of 16.1 ± 4.5 μM in RLM and 14.4 ± 0.6 μM in HLM. However, the metabolism of REP in RLM and HLM was not significantly altered by CEL (data not shown). Additionally, protein binding interactions between the two drugs in rat and human plasma were assessed. As shown in Figure 5, there were no significant differences in fractions of unbound drugs either alone or in combination, suggesting the minimal possibility of protein binding-based interactions between the two drugs.

Since oral AUC is calculated as F × D/CL (F, oral bioavailability; D, dose; CL, total clearance), an increase in F and/or decrease in CL result in an increase in AUC. Moreover, hepatic metabolism is the major elimination route for both REP and CEL that are drugs with a low hepatic extraction ratio. Thus, the inhibition of hepatic metabolism of the two drugs can reduce their hepatic first-pass effect (increase in F) and hepatic systemic clearance (decrease in CL), consequently leading to an increase in AUC. In our present in vitro metabolism study in RLM and HLM, as shown in Figure 4, the metabolism of REP was not significantly changed by CEL, while the metabolism of CEL was inhibited by REP with a mean IC_50_ of 16.1 μM in RLM and 14.4 μM in HLM. Assuming that the in vivo concentration levels of REP in the rat liver after oral dosing are high enough to inhibit the metabolism of CEL, the increased oral systemic exposure of CEL by concurrent administration of REP (AUC_last_ in Table 4) could be attributable to a reduction of hepatic first-pass effect and/or hepatic systemic clearance of CEL caused by the inhibitory activity of REP on the hepatic metabolism of CEL.

The present rat study highlighted the possibility for metabolism-based interactions between CEL and REP in clinical settings. As shown in Figure 4, REP significantly inhibited the metabolic reaction of CEL with comparable IC_50_ values between RLM and HLM. The *C*_max_ of REP administered orally to rats was reported to be 297 ± 103 ng/mL (dose: 0.4 mg/kg) in this study, as shown in Table 4, and 105.1 ± 30 ng/mL (dose: 0.5 mg/kg) in a previous study [35], which are roughly comparable to the reported *C*_max_ of 65.8 ± 30.1 ng/mL (dose: 4 mg; converted to 0.41 mg/kg in rats based on the human equivalent dose concept proposed by the FDA) in humans (FDA drug label information). Moreover, there were no significant differences in the unbound fraction of REP between rat and human plasma, as shown in Figure 5. Based on these findings, it is plausible that the increased systemic exposure of CEL by co-administration of REP in the present rat pharmacokinetic study could have some clinical relevance, depending on species differences in hepatic distribution profiles of REP between rats and humans.

Additionally, there have been no reported studies on the relationships between the AUC and toxicity of CEL. However, the FDA drug label of CEL indicates that the steady-state AUC of CEL is increased about 40% and 180% by mild (Child-Pugh Class A) and moderate (Child-Pugh Class B) hepatic impairment, respectively. Thus, the daily recommended dose of CEL should be reduced by approximately 50% in patients with moderate (Child-Pugh Class B) hepatic impairment. In our present study, the AUC_last_ of CEL in rats was observed to be 189 (ranging from 113 to 285) μg·min/mL after administration of CEL alone and 333 (ranging from 458) μg·min/mL after administration of CEL and REP in combination. If these rat data could be extrapolated to humans, it is plausible that careful toxicity monitoring and/or dose modification may be needed for the combined dose of CEL and REP in clinical practice. Despite intrinsic limitations associated with nonclinical studies, our present in vivo rat and in vitro HLM data warrant further clinical study on drug interactions between REP and CEL.

## 4. Conclusions

This study successfully developed a simple, sensitive, and validated HPLC-FL method for simultaneous determination of REP and CEL in rat plasma. The new bioanalytical method provided several merits, including good sensitivity, high extraction recovery, negligible matrix effect, and simplicity of sample preparation procedures. The application of this method in the study of pharmacokinetic interactions between REP and CEL revealed that the pharmacokinetics of oral CEL were significantly altered by concurrent administration with oral REP. Furthermore, an in vitro metabolism and protein binding study using HLM and human plasma highlighted the possibility of metabolism-based interactions between CEL and REP in clinical settings. Therefore, the bioanalytical method proposed herein could become a promising tool for preclinical pharmacokinetic studies and, by extension, clinical use after partial modification and validation.

## Figures and Tables

**Table 1:** Intra- and inter-day precision and accuracy of REP and CEL in rat plasma (*n* = 5). HQC: high quality control.

Nominal Concentration (ng/mL)	Precision (%)		Accuracy (%)	
	Intra-Day	Inter-Day	Intra-Day	Inter-Day
*Repaglinide (REP)*				
LLOQ (10)	2.69	6.21	112	101
LQC (30)	1.20	3.99	104	99.4
MQC (120)	1.16	0.70	102	101
HQC (1200)	0.79	1.43	99.2	99.8
*Celecoxib (CEL)*				
LLOQ (10)	4.06	8.30	111	103
LQC (30)	0.79	2.15	104	103
MQC (120)	1.19	2.18	100	102
HQC (1200)	1.55	1.63	98.6	100

**Table 2:** Recovery and matrix effect of REP, CEL, and IS in rat plasma (*n* = 5).

Nominal Concentration (ng/mL)	Recovery (%)	Matrix Effect (%)
*Repaglinide (REP)*		
LLOQ (10)	98.5 ± 5.3	101 ± 6
LQC (30)	98.6 ± 1.1	102 ± 4
MQC (120)	103 ± 2	95.6 ± 3.0
HQC (1200)	101 ± 1	92.1 ± 2.5
*Celecoxib (CEL)*		
LLOQ (10)	104 ± 3	97.6 ± 3.0
LQC (30)	102 ± 4	98.5 ± 3.5
MQC (120)	104 ± 2	93.8 ± 1.8
HQC (1200)	103 ± 2	90.7 ± 2.4
IS (Ketoconazole, 50)	96.7 ± 1.2	99.4 ± 1.3

**Table 3:** Stability (%) of REP and CEL in rat plasma (*n* = 5).

Nominal Concentration (ng/mL)	Bench-Top ^a^	Autosampler ^b^	Freeze‒Thaw ^c^	Long-Term ^d^
*Repaglinide (REP)*				
LLOQ (10)	102 ± 6	106 ± 3	106 ± 6.	96.4 ± 3.4
LQC (30)	92.7 ± 4.2	102 ± 4	98.1 ± 3.7	97.6 ± 3.7
MQC (120)	92.9 ± 1.4	104 ± 0	102 ± 2	95.1 ± 1.8
HQC (1200)	90.6 ± 0.7	101 ± 1	100 ± 1	93.3 ± 0.8
*Celecoxib (CEL)*				
LLOQ (10)	98.3 ± 2.4	95.2 ± 2.6	90.3 ± 2.4	92.7 ± 3.0
LQC (30)	89.2 ± 2.7	97.8 ± 1.8	97.9 ± 2.1	92.7 ± 3.5
MQC (120)	93.1 ± 2.2	103 ± 1	101 ± 2	92.9 ± 1.2
HQC (1200)	89.6 ± 1.9	101 ± 1	99.6 ± 0.4	91.7 ± 0.7

^a^ Room temperature for 3 h. ^b^ 10 °C for 1 day in the autosampler. ^c^ Three freezing and thawing cycles. ^d^ −20 °C for 30 days.

**Table 4:** Pharmacokinetic parameters of REP and CEL in rats after oral administration of 0.4 mg/kg REP and 2 mg/kg CEL either alone or in combination (*n* = 5).

Parameter	REP		CEL	
	Single	Combined	Single	Combined
AUC_last_ (×10^3^ ng∙min/mL)	21.1 ± 5.1	25.9 ± 2.8	189 ± 70	333 ± 89 *
AUC_inf_ (×10^3^ ng∙min/mL)	30.5 ± 5.5	35.4 ± 8.3	ND	ND
*C*_max_ (ng/mL)	297 ± 103	224 ± 102	741 ± 299	954 ± 196
*T*_max_ (min)	20 (20–45)	45 (20–60)	180 (30–240)	120 (45–360)
*t*_1/2_ (min)	374 ± 45	290 ± 123	ND	ND

* Significantly different from the single group (*p* < 0.05).

## Supplementary Materials

The following is available online at https://www.mdpi.com/1999-4923/11/8/382/s1, Table S1. pharmacokinetic parameters of intravenous repaglinide (REP) and celecoxib (CEL) reported in previous studies on rats.
