# Supplementary material for: Pharmacokinetic Evaluation of Metabolic Drug Interactions between Repaglinide and Celecoxib by a Bioanalytical HPLC Method for Their Simultaneous Determination with Fluorescence Detection
Source: Pharmaceutics. 2019 Aug 2;11(8):382. doi: 10.3390/pharmaceutics11080382 (PMC6723536; doi:10.3390/pharmaceutics11080382)
Supplement: Supplementary file 1 [file pharmaceutics-11-00382-s001.pdf]

pharmaceutics

Supplementary Materials: Pharmacokinetic Evaluation of Metabolic Drug Interactions between Repaglinide and Celecoxib by a Bioanalytical HPLC Method for Their Simultaneous Determination with Fluorescence Detection

Dong-Gyun Han, Jinsook Kwak, Seong-Wook Seo, Ji-Min Kim, Jin-Wook Yoo, Yunjin Jung, Yun-Hee Lee, Min-Soo Kim, Young-Suk Jung, Hwayoung Yun and In-Soo Yoon

Table S1. pharmacokinetic parameters of intravenous repaglinide (REP) and celecoxib (CEL) reported in previous studies on rats.

Parameter	REP	CEL
Dose (mg/kg)	0.2	1
AUC (×10^3 ng·min/mL)	38.8	129
CL (mL/min/kg)	5.2 ± 1.0	7.76
Dose excreted in urine (%)	0.08 (human, oral)	0.04
References	[1,2]	[3]

References

1. Choi, J.S., Choi, I., Choi, D.H. Effects of nifedipine on the pharmacokinetics of repaglinide in rats: Possible role of CYP3A4 and P-glycoprotein inhibition by nifedipine. *Pharmacol. Rep.* **2013**, *65*, 1422–1430.
2. van Heiningen, P.N., Hatorp, V., Kramer Nielsen, K., Hansen, K.T., van Lier, J.J., De Merbel, N.C., Oosterhuis, B., Jonkman, J.H. Absorption, metabolism and excretion of a single oral dose of (14)C-repaglinide during repaglinide multiple dosing. *Eur. J. Clin. Pharmacol.* **1999**, *55*, 521–525.
3. Paulson, S.K., Zhang, J.Y., Breau, A.P., Hribar, J.D., Liu, N.W., Jessen, S.M., Lawal, Y.M., Cogburn, J.N., Gresk, C.J., Markos, C.S., Maziasz, T.J., Schoenhard, G.L., Burton, E.G. Pharmacokinetics, tissue distribution, metabolism, and excretion of celecoxib in rats. *Drug Metab. Dispos.* **2000**, *28*, 514–521.
